# A population-based cohort study on adherence to practice guidelines for adjuvant chemotherapy in colorectal cancer

**DOI:** 10.1186/1471-2407-14-948

**Published:** 2014-12-13

**Authors:** Elinor Bexe Lindskog, Katrín Ásta Gunnarsdóttir, Kristoffer Derwinger, Yvonne Wettergren, Bengt Glimelius, Karl Kodeda

**Affiliations:** Department of Surgery, Institute of Clinical Sciences, Sahlgrenska Academy, University of Gothenburg, Gothenburg, Sweden; Regional Cancer Centre (West), Western Sweden Health Care Region, Gothenburg, Sweden; Department of Radiology, Oncology and Radiation Science, Uppsala University, Uppsala, Sweden; Department of Surgery, Sahlgrenska University Hospital, 416 85 Göteborg, Sweden

**Keywords:** Registries, Chemotherapy adjuvant, Colonic neoplasms, Rectal neoplasms, Practice guidelines

## Abstract

**Background:**

The value of adjuvant chemotherapy in colorectal cancer is well studied, and guidelines have been established. Little is known about how treatment guidelines are implemented in the everyday clinical setting.

**Methods:**

This national population-based study on nearly 34,000 patients with colorectal cancer evaluates the adherence to present clinical guidelines for adjuvant chemotherapy. Virtually all patients with colorectal cancer in Sweden during the years 2007–2012 and data from the Swedish Colorectal Cancer Registry were included.

**Results:**

In colon cancer stage III, adherence to national guidelines was associated with lower age, presence of multidisciplinary team (MDT) conference, low co-morbidity, and worse N stage. The MDT forum also affected whether or not high-risk stage II colon cancer patients were considered for adjuvant chemotherapy. Rectal cancer patients both in stage II and III were considered for adjuvant chemotherapy less often than colon cancer patients, but the same factors influenced the decision. Adjuvant chemotherapy was started later than eight weeks after surgery in 30% of colon cancer patients and in 38% of rectal cancer patients.

**Conclusions:**

In Sweden, the adherence to national guidelines for adjuvant chemotherapy in colon cancer stage III is acceptable in younger and healthier patients. MDT conferences are of major importance and affect whether patients are recommended for adjuvant chemotherapy. Special consideration needs to be given to certain subgroups of patients, particularly older patients and patients with poorly differentiated tumors. There is a need to shorten the waiting time until start of chemotherapy.

**Electronic supplementary material:**

The online version of this article (doi:10.1186/1471-2407-14-948) contains supplementary material, which is available to authorized users.

## Background

In Sweden, almost 6000 patients are diagnosed annually with colorectal cancer (CRC), which is the third most common cancer in the world [1]. Surgery offers the best chance for curing CRC, but adjuvant chemotherapy can further improve survival. While international and national guidelines regarding indications for adjuvant chemotherapy in CRC have been established, few population-based studies have evaluated adherence to practice guidelines. Staging and treatment have evolved in recent decades. In Sweden there are nationally accepted guidelines, which are currently under revision [2]. Since 2008, the guidelines have recommended that patients younger than 76 years of age with stage III colon cancer should be considered for six months of 5-FU-calciumfolinate or capecitabine alone or in combination with oxaliplatin. High-risk stage II colon cancer may be eligible for treatment, as in stage III. Adjuvant chemotherapy is not recommended for rectal cancer stage II or III.

The European Society for Medical Oncology (ESMO) recently published guidelines for CRC [3–5]. These guidelines recommend adjuvant chemotherapy for high-risk stage II and stage III colon cancer; although it is recognized that there is less scientific evidence, it is also written that patients with high-risk stage II and stage III rectal cancer could receive adjuvant chemotherapy as in colon cancer. However, at the 2013 European Registration of Cancer Care (EURECCA) consensus conference, minimal or no consensus was reached regarding adjuvant chemotherapy for rectal cancer [6]. The guidelines from the American National Comprehensive Cancer Network (NCCN) are consistent with the ESMO guidelines, but they also include the possibility of adjuvant chemotherapy for patients with low-risk stage II disease [7, 8].

Some studies on adherence to clinical guidelines have been conducted, including one large study from the United States that presents a stage-dependent difference in adherence in which high-risk stage II colon cancer had the lowest correspondence [9]. Other studies have suggested an association between older age and lower adherence to guidelines, especially regarding the prescription of oxaliplatin [10–13]. In contrast, one recent study found high compliance levels in elderly patients; however, patients defined as elderly were younger than in the previously mentioned studies [14].

To obtain a population-based patient cohort is difficult, and when selected centers or local regions with low coverage of the population are used, there is a risk of selection bias. Sweden has the unique opportunity of performing truly national population-based studies; nearly all patients with CRC are included in a quality control registry. The main purposes of the registry are to audit management and outcome, report data for quality improvements, and provide valid data for research. The aim of this study was to evaluate adherence to national guidelines on adjuvant chemotherapy.

## Methods

### Data and cohort construction

The Swedish Colorectal Cancer Registry (SCRCR) captures at least 99% of all patients diagnosed with CRC in Sweden [15, 16]. The registry has been validated against medical records for a full-year cohort, showing 94–97% agreement on six variables, and a study on the validity of the registry’s first three years deemed it as “good” [17].

The inclusion criterion for this study was patients registered in the SCRCR from 1 January 2007 to 31 December 2012, and the primary outcome of interest was *planned adjuvant chemotherapy*. Patients in stage II or III are eligible for adjuvant chemotherapy in different guidelines; thus, patients with stage I or IV or with no stage listed were excluded. In addition, patients who underwent local excision or who had no surgical resection were excluded in order to ensure a true stage classification. Since 2009, the department responsible for oncological treatment has also reported data on started chemo- and radiotherapy; therefore, the secondary outcome of interest was *started adjuvant chemotherapy*. Data on patients with records from 1 January 2009 to 31 December 2012 were then obtained from the oncology database.

A total of 1086 patients had two or more registered occurrences of CRC, which were counted as one. Patients were restaged according to the 7th edition of the tumor node metastasis (TNM) staging system of the International Union against Cancer/American Joint Committee on Cancer using pathological data of the number of positive lymph nodes. Tumor deposits or satellites in the lymph drainage area of pericolorectal adipose tissue are classified as N1c according to the 7th edition; however, they were not recorded in the SCRCR before 2011 and are disregarded here and classified as N0. Histological grading was regarded according to the new dichotomized scale: low grade (G1–G2) and high grade (G3–G4). Patients with stage II disease were subgrouped according to high or low risk. Patients included in the high-risk population were those with an emergency intestinal occlusion or perforation, lymph node sampling less than 12, T4 tumor, poorly differentiated tumor (G3–G4), and vascular or perineural invasion. However, lymphatic invasion is not reported in the SCRCR, and information about vascular and perineural invasion is sometimes lacking in the pathology report.

This study was approved by the regional ethical review board in Gothenburg, (Decision Number 072-13). The data analysed in this study are not publicly accessible. After approval from the regional ethical review board, permission was granted from the steering group of the SCRCR for extraction of registry-data. The SCRCR data-set is continuously updated and data for this study was extracted on May 24th 2013.

### Statistical analyses

The data were summarized using contingency tables. All analyses were conducted separately for colon cancer and rectal cancer. For the subgroup of patients with stage III colon cancer, univariate logistic regression was applied to assess the putative relation of classical risk factors on the outcome, quantified in terms of 95% confidence intervals.

In order to adjust for possible confounding, the resulting factors of interest were included in a multivariate logistic regression analysis. First-level interactions of gender and age against all other covariates were each entered into the model separately; none was found to be significant. Goodness of fit of the final model was assessed using the Hosmer–Lemeshow chi-square statistics [18]. Confidence intervals and Wald tests were used to evaluate significance in the multivariate analyses.

All analyses were carried out using the R 2.15.1 software [19].

## Results

**Figure 1:**
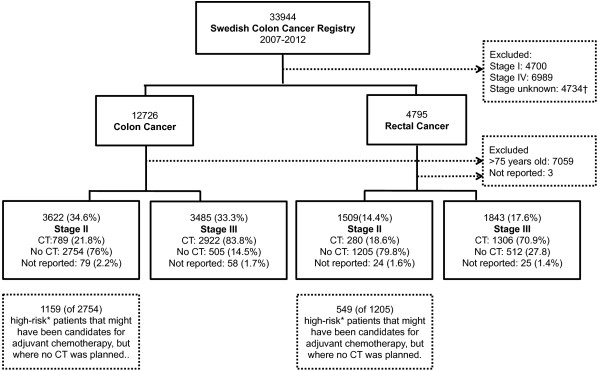
**Flow chart of the study. Abbreviation: CT = chemotherapy.** ^*^High-risk patients: clinical presentation with intestinal occlusion or perforation, lymph nodes sampling <12, pT4, poorly differentiated tumour (G3-G4), vascular or perineural invasion. † Extent of surgery; Local excision (n = 334), no surgery (n = 1243), surgery (n = 2224) and missing (n = 933).

During the six-year period, 33,944 patients were included in the SCRCR, of which17,521 were in stage II or III (Figure 1). Of these patients, 7602 were older than 75 years of age and were excluded from selected analyses. The demographics and characteristics of the patients are reported in Table 1. Of the 10,459 patients younger than 76 years of age, 5297 (50.6%) were planned for adjuvant chemotherapy.

**Table 1:** **Demographics and clinical characteristics**

	Colon		Rectum		Total	
	(N)		(N)		(N)	
Age (years (%))						
≤75	7107	(55.8)	3352	(69.9)	10459	(59.7)
>75	5616	(44.1)	1443	(30.1)	7059	(40.3)
Missing data	3	(0.0)	-		3	(0.0)
Gender (%)						
Male	6179	(48.6)	2854	(59.5)	9033	(51.6)
Female	6547	(51.4)	1941	(40.5)	8488	(48.4)
Elective surgery (%)						
Yes	10306	(81.0)	4731	(98.7)	15037	(85.8)
No	2419	(19.0)	60	(1.3)	2479	(14.1)
Missing data	1	(0.0)	4	(0.1)	5	(0.0)
Stage* (%)						
II (T3, T4, N0)	6842	(53.8)	2211	(46.1)	9053	(51.7)
III (Any T, N1, N2)	5884	(46.2)	2584	(53.9)	8468	(48.3)
Mucinous (%)						
Yes	2583	(20.3)	651	(13.6)	3234	(18.5)
No	8863	(69.6)	3593	(74.9)	12456	(71.1)
Missing data	1280	(10.0)	551	(11.5)	1831	(10.0)
Examined lymph nodes (%)						
<12	1936	(15.2)	1171	(24.4)	3107	(17.7)
≥12	10644	(83.6)	3571	(74.5)	14215	(81.1)
Missing data	146	(1.1)	53	(1.1)	199	(1.1)
Tumor differentiation* (%)						
Low grade (G1, G2)	9268	(72.8)	3830	(79.9)	13098	(74.8)
High grade (G3, G4)	3004	(23.6)	746	(15.6)	3750	(21.4)
Not indicated	390	(3.1)	193	(4.0)	583	(3.3)
Missing data	64	(0.5)	26	(0.5)	90	(0.5)
ASA^†^ (%)						
1	1842	(14.5)	1002	(20.9)	2844	(16.2)
2	6443	(50.6)	2620	(54.6)	9063	(51.7)
3	3719	(29.2)	1010	(21.1)	4729	(27.0)
4	362	(2.8)	66	(1.4)	428	(2.4)
5	4	(0.0)	0	(0.0)	4	(0.0)
Missing data	356	(2.8)	97	(2.0)	453	(2.6)
Region of treatment (%)						
Northern	1164	(9.1)	444	(9.3)	1608	(9.2)
Uppsala/Örebro	2873	(22.6)	1110	(23.1)	3983	(22.7)
Stockholm/Gotland	2380	(18.7)	868	(18.1)	3248	(18.5)
Western	2311	(18.2)	909	(19.0)	3220	(18.4)
South-eastern	1533	(12.0)	558	(11.6)	2091	(11.9)
Southern	2464	(19.4)	906	(18.9)	3370	(19.2)
Missing data	1	(0.0)	-		1	(0.0)

Stage II-III colorectal cancer patients operated with resection of the tumor during 2007 to 2012. Data from the Swedish colorectal cancer registry.

*TNM, 7^th^ edition from UICC/AJCC (Union for International Cancer Control/American Joint Committee on Cancert). ^†^American Society of Anesthesiologists Physical Status Classification System.

### Colon cancer stage III

Guidelines recommend adjuvant chemotherapy in colon cancer stage III, and of 3485 patients younger than 76 years of age, 2922 (83.8%) were planned for this treatment (Figure 1). Factors associated with treatment were age (p < 0.01), comorbidity (p < 0.01), and N stage (p < 0.01) (Table 2). Discussing patients in an MDT conference (p < 0.01) also affected whether adjuvant chemotherapy was planned; it was planned in 81.7% of patients younger than 76 years of age, ASA 1-2, who were not discussed and in 90.6% in patients who were discussed. Patients younger than 60 years of age were evaluated in MDT conferences in 82.4% of the cases, as were 79.4% of patients 60–75 years of age and 68.4% of patients older than 75 years of age. Further subgroup analyses are presented in Additional file 1: Table S1.

**Table 2:** **Patients planned for adjuvant chemotherapy, younger than 76 years with stage III colon cancer by patient and health-care region (n = 3427)**

	Univariate		Multivariate		
	OR	95% CI	OR	95% CI	P ^‡^
**Gender**					
Male	1.00		1.00		
Female	1.36	1.13-1.65	1.29	1.04-1.61	0.021
**Age (years)**					
60-75	1.00		1.00		
<60	2.65	2.02-3.55	2.14	1.57-2.98	<0.01
ASA classification*					
3-4	1.00		1.00		
1-2	4.31	3.51-5.28	4.26	3.14-5.33	<0.01
**N-stage** ^**†**^					
1a	1.00		1.00		
1b	1.29	1.01-1.66	1.20	0.92-1.57	
2	1.64	1.29-2.08	1.62	1.24-2.12	<0.01
**Tumor differentiation** ^**†**^					
Low-grade (G1, G2)	1.00		1.00		
High-grade (G3, G4)	0.94	0.76-1.17	0.84	0.66-1.08	0.17
**Planned surgery**					
Yes	1.00		1.00		
No	0.79	0.63-0.99	0.83	0.64-1.09	0.18
**Multidisciplinary conference**					
No	1.00		1.00		
Yes	1.78	1.44-2.21	1.83	1.41-2.37	<0.01
**Region**					
Northern	1.00		1.00		
Stockholm-Gotland	1.29	0.89-1.84	0.96	0.63-1.46	
Uppsala-Örebro	1.45	1.02-2.05	1.14	0.75-1.71	
Southeastern	1.10	0.74-1.63	0.89	0.56-1.40	
Southern	1.33	0.92-1.89	1.17	0.76-1.79	
Western	1.16	0.82-1.64	0.95	0.63-1.42	0.67

Complete case univariate and multivariate logistic regression.

*Abbreviation*: OR, Odds Ratio. *American Society of Anesthesiologists Physical Status Classification System. ^†^TNM, 7^th^ edition from UICC/AJCC (Union for International Cancer Control/American Joint Committee on Cancert). ^‡^Statistical method; Wald test.

### Colon cancer stage II

**Figure 2:**
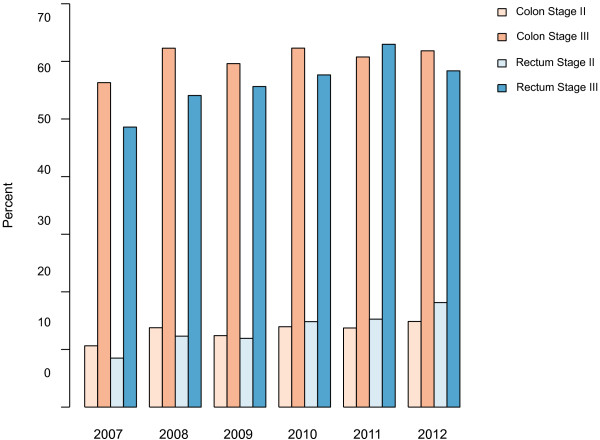
**Proportion of patients where adjuvant chemotherapy was planned per year for each combination of stage and site (n = 16 690).** Stratified multivariate analyses where year is entered as a continuous covariate in a model, which also adjusts for age, and sex indicate an increasing trend for adjuvant chemotherapy in all four groups (p < 0.05).

As discussed in the background section, patients in stage II also may be recommended adjuvant chemotherapy. Colon cancer stage II patients younger than 76 years of age were planned for adjuvant chemotherapy in 789 (21.8%) of the cases; of those 722 (91.5%) were high risk and 67 (8.5%) were low risk (Figure 1). There was an increase in patients planned for adjuvant chemotherapy over time (Figure 2). Patients meeting at least one high-risk criterion and not planned for adjuvant chemotherapy numbered 1159 (Figure 1). The proportion of high-risk patients considered for adjuvant chemotherapy was lower at older ages, in the presence of comorbidity, and in the absence of an MDT conference (Additional file 1: Table S1).

Of the high-risk stage II patients, 33.3% met more than one high-risk criterion. There was a high proportion of patients planned for adjuvant chemotherapy among patients with the high-risk criterion pT4 (63.5%) and almost half of the patients were planned for chemotherapy if vascular or perineural invasion was present (Table 3).

**Table 3:** **Stage II, colon and rectal cancer, younger than 76 years – risk factors and planned adjuvant chemotherapy**

	Colon		Rectum	
	Planned CT	No CT	Planned CT	No CT
**Total number of patients**	663	1159	204	549
pT4	363 (63.5)	209 (36.5)	57 (45.6)	68 (54.4)
N (%)				
Poorly differentiated	201 (32.7)	414 (67.3)	49 (29.3)	118 (70.7)
N (%)				
Vascular invasion	187 (48.4)	199 (51.6)	73 (32.6)	151 (67.4)
N (%)				
Intestinal occlusion	127 (33.7)	250 (66.3)	2 (33.3)	4 (66.7)
N (%)				
Lymph node sampling <12, N (%)	116 (27.2)	310 (72.8)	85 (23.2)	281 (76.8)
Perineural invasion	90 (45.9)	106 (54.1)	60 (37.7)	99 (62.3)
N (%)				
Intestinal perforation	27 (37.5)	45 (62.5)	24 (37.5)	40 (62.5)
N (%)				

More than one criterion can apply for each patient.

*Abbreviation*: CT, Chemotherapy.

### Rectal cancer stage II-III

Of 1843 patients with stage III rectal cancer, 1306 (70.9%) were planned for adjuvant chemotherapy (Figure 1), as were 280 (18.6%) of 1509 patients with stage II rectal cancer. These proportions were lower compared to colon cancer (Figure 1). The proportions of patients considered for adjuvant chemotherapy increased from 2007 to 2012 in both stage II and III (Figure 2).

As in colon cancer the highest proportion of patients in stage II with a high-risk criterion planned for adjuvant chemotherapy was seen among patients with a pT4 tumor (45.6%, Table 3).

### Oncology dataset 2009–2012

Of 4272 patients with CRC planned for adjuvant chemotherapy between 2009 and 2012, oncology data was reported in 3985 (93.3%) cases. In colon cancer, adjuvant chemotherapy was started in 91.8% (n = 2812) of resected patients planned for adjuvant chemotherapy. The corresponding figure for rectal cancer was 76.6% (n = 1173).

Where oncology data indicated chemotherapy for a colon cancer (n = 2595), 5-FU or capecitabine was prescribed as a single therapy in about half of the cases in stage II and stage III N1, and in 30.9% of stage III N2 cases (Table 4). A combination with oxaliplatin was prescribed at highest proportion to stage III N2 (63.3%) and N1 (48.6%), see Table 4. The proportion of patients treated with oxaliplatin was age dependent, and in stage III N2 82.9% of patients younger than 70 years received the combination with oxaliplatin, compared to 63.5% if stage III N1. Adjuvant chemotherapy was started within six weeks of surgery in 18.2% of the cases, more than eight weeks after surgery in 30.1%, and more than 12 weeks after surgery in 4.1%.

**Table 4:** **Started postoperative chemotherapy stage II-III**

	Stage II		Stage III	
	Low risk (%)	High risk (%)	N1 (%)	N2 (%)
**Colon cancer**	107	539	1425	1088
Capecitabine/5-FU	51 (47.7)	296 (54.9)	662 (46.4)	336 (30.9)
Capecitabine/5-FU+	42 (39.2)	209 (38.8)	693 (48.6)	689(63.3)
Oxaliplatin				
Other combinations	14 (13.1)	34 (6.3)	70 (4.9)	63 (5.8)
**Rectal cancer**	135	258	556	471
Capecitabine/5-FU	72 (53.3)	142 (55.0)	294 (52.9)	168 (35.7)
Capecitabine/5-FU+	33 (24.4)	66 (25.6)	203 (36.5)	245 (52.0)
Oxaliplatin				
Other combinations	28 (20.7)	50 (19.4)	59 (10.6)	58 (12.3)

*Abbreviation*: 5-FU, 5-fluorouracil.

Of the 922 patients with rectal cancer who received adjuvant chemotherapy, 239 patients had received preoperative chemotherapy. Of those patients, 211 (88.3%) received long-course radiotherapy, and 123 (51.5%) had a primarily unresectable tumor. In comparison, among the 1560 patients with rectal cancer who did not receive adjuvant chemotherapy, 248 patients received preoperative long-course chemoradiation, and 121 had a primarily unresectable tumor. Thus, 211 (46.0%) of 459 patients who received preoperative chemoradiation started adjuvant chemotherapy postoperatively. Short-course radiotherapy (5x5Gy) was given to 1997 patients, 522 (26.1%) of these received adjuvant chemotherapy. 5-FU or capecitabine alone was prescribed to more than half of the cases in stage II and stage III N1 and in 35.7% of the cases in stage III N2. As in colon cancer, combination treatment was prescribed in highest proportion to stage III N2 (52.0%), see Table 4. Adjuvant chemotherapy was started within six weeks of surgery in 16.9% of the cases, more than eight weeks after surgery in 37.0%, and more than 12 weeks after surgery in 4.7%.

## Discussion

In this population-based dataset covering over 99% of patients diagnosed with CRC in Sweden, the adherence to national guidelines for adjuvant chemotherapy in colon cancer stage III was high in younger and healthier patients. However, the adherence was considerably lower in some subgroups of patients.

In recent years, with a multimodal approach to CRC treatment, the importance of MDT conferences has increased [6]. In this study, patients with both high-risk stage II and stage III colon cancer were planned for adjuvant chemotherapy considerably more often when they were discussed in a postoperative MDT conference (stage III, p < 0.01, see Table 2). There was an age-dependent difference in the proportion of patients brought up at MDT conferences, indicating a tendency toward leaving out MDT conferences in the elderly and comorbid populations. However, the differences persisted after correction for age, comorbidities, and N-stage. The impact of MDT conferences affecting the proportion of patients receiving adjuvant chemotherapy is supported by other studies, and some studies also show a correlation between MDT and better survival [20, 21].

Nodal stage is the main factor determining whether guidelines recommend adjuvant chemotherapy for CRC patients. As a prognostic factor, the number of positive lymph nodes is important in both colon and rectal cancer in stage III [22]. This is reflected in adherence to guidelines, with an increased proportion of patients with a more advanced N stage being recommended for chemotherapy (Table 2). In contrast, tumor differentiation, which is a stage-independent prognostic factor in CRC, does not affect whether patients are planned for adjuvant chemotherapy [23, 24]. Emergency surgery did not either affect whether patients were planned for adjuvant chemotherapy, even though this is one of the high-risk factors and an association with worse cancer-specific long-term survival has been shown in several studies [15, 25].

Presence of comorbidities was another main factor influencing whether patients were planned for adjuvant chemotherapy, and patients with ASA 1–2 were four times more likely to be recommended chemotherapy than patients with ASA 3–4. Age was another factor influencing adjuvant chemotherapy. Even when corrected for comorbidities included in the ASA classification, patients younger than 60 years of age were planned for chemotherapy more than twice as often as patients 60–75 years of age. Our results are consistent with the results of other registry studies, which also reported lower adherence to treatment guidelines in older patients, independent of pre-existing comorbidities [9, 12, 26].

Swedish guidelines do not recommend adjuvant chemotherapy for rectal cancer. However, adjuvant chemotherapy was planned in about 70% of patients with stage III rectal cancer, and it was started in three out of four patients (2009–2012). The data show an overtreatment in regard to national guidelines that increased over time and likely reflect that many physicians follow international guidelines. However, adjuvant chemotherapy for rectal cancer is an extremely controversial issue due to the lack of clear evidence from randomized trials [27–30]. Its use is particularly controversial in patients who have had preoperative chemoradiotherapy; in addition to the EORTC 22921 study, three other European randomized trials have not been able to detect a significant survival gain [27, 31–33]. It is presently an open question what the updated Swedish guidelines will recommend. It is possible that rectal cancers should not be handled homogeneously; rectal tumors in the upper intraperitoneal third could be handled as colon cancers, as opposed to tumors arising extraperitoneally [34]. In light of this lack of scientific knowledge, it is surprising that approximately half of patients who received preoperative chemoradiotherapy because of a locally advanced tumor continued with postoperative adjuvant therapy. The international guidelines, along with a belief that it should work, have had a great impact [3–5, 7, 8].

Most patients with rectal cancer who received preoperative chemoradiation therapy had locally advanced tumors. Therefore, the main reason to start chemotherapy preoperatively was probably to potentiate the radiotherapy effect, in which case capecitabine alone is presently used. The concept of providing adequate systemic therapy upfront is currently being explored in trials [27, 35].

In colon cancer stage III the combination of 5-FU with oxaliplatin is associated with better disease-free survival and overall survival [36–38]. However, the oxaliplatin combination-therapy is associated with more side effects and might not be applicable to all patients [38]. The number of patients treated with combination-therapy might be considered low (Table 4), it was however age-dependent and in patients younger than 70 the proportion of patients prescribed a combination-therapy was considerably higher. Timing of chemotherapy is another topic of discussion. Studies have shown value in an early start of adjuvant chemotherapy; starting chemotherapy later than 12 weeks after surgery is of questionable value, although some studies have indicated a survival benefit even with a late start. [39, 40] In the Swedish guidelines, there are no specific recommendations for the timing of adjuvant chemotherapy; however, several regions recommend that every effort should be made to start as early as possible, and no later than eight weeks. There is no scientific rationale for eight weeks, but it should be remembered that all colon cancer trials that have shown a significant survival gain required that therapy should start within 5–6 weeks. The European guidelines recommend as early a start as possible, from the third week up to a maximum of 8–12 weeks after surgery. In the present study cohort, one-third of the patients started their adjuvant chemotherapy later than eight weeks postoperatively.

The benefit from adjuvant chemotherapy on a group level and the absolute risk reduction of developing metastatic disease is lower in stage II than in stage III cancer. To better identify patients who might benefit from chemotherapy in stage II, patients need to be separated into high- and low-risk groups. ESMO and NCCN guidelines now recommend adjuvant chemotherapy for high-risk stage II cancer. In this study, there was an increase over time in the proportion of patients with stage II disease considered for chemotherapy in both rectal and colon cancer (Figure 2). Still there was a large portion of patients not considered for adjuvant chemotherapy even though one or several high-risk criteria were fulfilled. As mentioned previously, conducting an MDT conference is one of the main factors influencing whether patients with stage II are planned for adjuvant chemotherapy. However, with improved surgical techniques and pathological examinations of surgical specimens, stage migration is a likely factor affecting changes to a better prognosis in stages II and III [41]. Thus, re-evaluation of some of the high-risk criteria in stage II is an important topic.

This study has several limitations. The data were not consistently validated, and information bias is a possibility. However, previous validations showed fair agreement with medical records, and we have no reason to believe this has changed. Most of our statistical calculations were carried out on data for planned chemotherapy and some were carried out on initiated treatment. Unfortunately, the SCRCR does not collect data on duration or compliance of chemotherapy regimens. The main strengths of the study are the sheer size and the fact that it is based on the entire Swedish population, and therefore, truly population based. Another strength is the ability to account for missing data.

## Conclusions

Although the general adherence levels to present national practice guidelines are acceptable in some aspects, all patients should be discussed in MDT conferences, as this factor seems to affect the proportion of patients considered for adjuvant chemotherapy, even in cases without significant comorbidities. Special consideration should be given to patients over the age of 60 and to patients with poorly differentiated tumors.

To possibly improve the outcome of, or at least make the best of, a given treatment, the time until the beginning of the therapy needs to be reduced. It has been hypothesized that an important reason for lack of a clear benefit in rectal as opposed to colon cancer is the inability to initiate the adjuvant chemotherapy early enough [42].

## Electronic supplementary material

Additional file 1: Table S1: Subgroup analyses in colon cancer stage II-III (n=12,726), 413 patients excluded due to missing data on planned adjuvant therapy. Patients remaining are 12,313. (DOC 92 KB)

## References

[1] Jemal A, Bray F, Center MM, Ferlay J, Ward E, Forman D (2011). Global cancer statistics. CA Cancer J Clin.

[2] **Kolorektal cancer: Nationellt vårdprogram: 2008 (in English: Colorectal cancer: National treatment plan: 2008)** 2008.http://www.cancercentrum.se/PageFiles/2823/Kolorektal%20cancer%20nya_vardprogrammet_081120%20(1).pdf

[3] Schmoll HJ, Van Cutsem E, Stein A, Valentini V, Glimelius B, Haustermans K, Nordlinger B, van de Velde CJ, Balmana J, Regula J, Nagtegaal ID, Beets-Tan RG, Arnold D, Ciardiello F, Hoff P, Kerr D, Kohne CH, Labianca R, Price T, Scheithauer W, Sobrero A, Tabernero J, Aderka D, Barroso S, Bodoky G, Douillard JY, El Ghazaly H, Gallardo J, Garin A, Glynne-Jones R (2012). ESMO Consensus Guidelines for management of patients with colon and rectal cancer. A personalized approach to clinical decision making. Ann Oncol.

[4] Glimelius B, Tiret E, Cervantes A, Arnold D (2013). Rectal cancer: ESMO Clinical Practice Guidelines for diagnosis, treatment and follow-up. Ann Oncol.

[5] Labianca R, Nordlinger B, Beretta GD, Mosconi S, Mandala M, Cervantes A, Arnold D (2013). Early colon cancer: ESMO Clinical Practice Guidelines for diagnosis, treatment and follow-up. Ann Oncol.

[6] van de Velde CJ, Boelens PG, Borras JM, Coebergh JW, Cervantes A, Blomqvist L, Beets-Tan RG, van den Broek CB, Brown G, Van Cutsem E, Espin E, Haustermans K, Glimelius B, Iversen LH, van Krieken JH, Marijnen CA, Henning G, Gore-Booth J, Meldolesi E, Mroczkowski P, Nagtegaal I, Naredi P, Ortiz H, Pahlman L, Quirke P, Rodel C, Roth A, Rutten H, Schmoll HJ, Smith JJ (2014). EURECCA colorectal: Multidisciplinary management: European consensus conference colon & rectum. Eur J Cancer.

[7] **NCCN Clinical practice Guidelines in Oncology: Colon Cancer** [http://www.nccn.org/professionals/physician_gls/pdf/colon.pdf] 10.6004/jnccn.2009.0056 19755046

[8] **NCCN Clinical Practice Guidelines in Oncology: Rectal Cancer** [http://www.nccn.org/professionals/physician_gls/pdf/rectal.pdf] 10.6004/jnccn.2009.0057 19755047

[9] Chagpar R, Xing Y, Chiang YJ, Feig BW, Chang GJ, You YN, Cormier JN (2012). Adherence to stage-specific treatment guidelines for patients with colon cancer. J Clin Oncol.

[10] Ayanian JZ, Zaslavsky AM, Fuchs CS, Guadagnoli E, Creech CM, Cress RD, O’Connor LC, West DW, Allen ME, Wolf RE, Wright WE (2003). Use of adjuvant chemotherapy and radiation therapy for colorectal cancer in a population-based cohort. J Clin Oncol.

[11] Abraham A, Habermann EB, Rothenberger DA, Kwaan M, Weinberg AD, Parsons HM, Gupta P, Al-Refaie WB (2013). Adjuvant chemotherapy for stage III colon cancer in the oldest old: Results beyond clinical guidelines. Cancer.

[12] Schrag D, Cramer LD, Bach PB, Begg CB (2001). Age and adjuvant chemotherapy use after surgery for stage III colon cancer. J Natl Cancer Inst.

[13] van Gils CW, Koopman M, Mol L, Redekop WK, Uyl-de Groot CA, Punt CJ (2012). Adjuvant chemotherapy in stage III colon cancer: guideline implementation, patterns of use and outcomes in daily practice in The Netherlands. Acta Oncol.

[14] O’Grady MA, Slater E, Sigurdson ER, Meropol NJ, Weinstein A, Lusch CJ, Sein E, Keeley P, Miller B, Engstrom PF, Cohen SJ (2011). Assessing compliance with national comprehensive cancer network guidelines for elderly patients with stage III colon cancer: the Fox Chase Cancer Center Partners’ initiative. Clin Colorectal Cancer.

[15] Kodeda K, Nathanaelsson L, Jung B, Olsson H, Jestin P, Sjovall A, Glimelius B, Pahlman L, Syk I (2013). Population-based data from the Swedish Colon Cancer Registry. Br J Surg.

[16] Pahlman L, Bohe M, Cedermark B, Dahlberg M, Lindmark G, Sjodahl R, Ojerskog B, Damber L, Johansson R (2007). The Swedish rectal cancer registry. Br J Surg.

[17] Jorgren F, Johansson R, Damber L, Lindmark G (2013). Validity of the Swedish Rectal Cancer Registry for patients treated with major abdominal surgery between 1995 and 1997. Acta Oncol.

[18] David W, Hosmer SL (2000). Applied Logistic Regression.

[19] **R:A language and enviroment for statistical computing. R Foundation for Statistical Computing, Vienna, Austria** [http://www.R-project.org/]

[20] MacDermid E, Hooton G, MacDonald M, McKay G, Grose D, Mohammed N, Porteous C (2009). Improving patient survival with the colorectal cancer multi-disciplinary team. Colorectal Dis.

[21] Du CZ, Li J, Cai Y, Sun YS, Xue WC, Gu J (2011). Effect of multidisciplinary team treatment on outcomes of patients with gastrointestinal malignancy. World J Gastroenterol.

[22] Resch A, Langner C (2013). Lymph node staging in colorectal cancer: Old controversies and recent advances. World J Gastroenterol.

[23] Derwinger K, Kodeda K, Bexe-Lindskog E, Taflin H (2010). Tumour differentiation grade is associated with TNM staging and the risk of node metastasis in colorectal cancer. Acta Oncol.

[24] Compton CC (2003). Colorectal carcinoma: diagnostic, prognostic, and molecular features. Mod Pathol.

[25] Wong SK, Jalaludin BB, Morgan MJ, Berthelsen AS, Morgan A, Gatenby AH, Fulham SB (2008). Tumor pathology and long-term survival in emergency colorectal cancer. Dis Colon Rectum.

[26] Allemani C, Rachet B, Weir HK, Richardson LC, Lepage C, Faivre J, Gatta G, Capocaccia R, Sant M, Baili P, Lombardo C, Aareleid T, Ardanaz E, Bielska-Lasota M, Bolick S, Cress R, Elferink M, Fulton JP, Galceran J, Gozdz S, Hakulinen T, Primic-Zakelj M, Rachtan J, Diba CS, Sanchez MJ, Schymura MJ, Shen T, Tagliabue G, Tumino R, Vercelli M (2013). Colorectal cancer survival in the USA and Europe: a CONCORD high-resolution study. BMJ Open.

[27] Bosset JF, Calais G, Mineur L, Maingon P, Stojanovic-Rundic S, Bensadoun RJ, Bardet E, Beny A, Ollier JC, Bolla M, Marchal D, Van Laethem JL, Klein V, Giralt J, Clavere P, Glanzmann C, Cellier P, Collette L (2014). Fluorouracil-based adjuvant chemotherapy after preoperative chemoradiotherapy in rectal cancer: long-term results of the EORTC 22921 randomised study. Lancet Oncol.

[28] Glimelius B (2010). Adjuvant chemotherapy in rectal cancer–an issue or a nonissue?. Ann Oncol.

[29] Bujko K, Glynne-Jones R, Bujko M (2010). Adjuvant chemotherapy for rectal cancer. Ann Oncol.

[30] Valentini V, van Stiphout RG, Lammering G, Gambacorta MA, Barba MC, Bebenek M, Bonnetain F, Bosset JF, Bujko K, Cionini L, Gerard JP, Rodel C, Sainato A, Sauer R, Minsky BD, Collette L, Lambin P (2011). Nomograms for predicting local recurrence, distant metastases, and overall survival for patients with locally advanced rectal cancer on the basis of European randomized clinical trials. J Clin Oncol.

[31] Cionini L, Sainato A, De Paoli A (2010). Final results of randimized trial on adjuvant chemotherapy after postoperative chemoradiation in rectal cancer.

[32] Breugom A, van den Broek C, van Gijn W: **The value of adjuvant chemotherapy for rectal cancer ptients after preoperative radiotherapy or chemoradiation and TME-surgery. Results of the PROCTOR/SCRIPT study.** *Eur J Cancer* 2013.,**49**(S3)**:** The European Cancer Congress 2013, Amsterdam

[33] Glynne-Jones R, Counsell N, Quirke P, Mortensen N, Maraveyas A, Meadows HM, Ledermann J, Sebag-Montefiore D: C (2014). Results of a randomised phase III trial in locally advanced rectal cancer after neoadjuvant chemoradiation randomising postoperative adjuvant capecitabine plus oxaliplatin (Xelox) versus control. Ann Oncol.

[34] Tiselius C, Gunnarsson U, Smedh K, Glimelius B, Pahlman L (2013). Patients with rectal cancer receiving adjuvant chemotherapy have an increased survival: a population-based longitudinal study. Ann Oncol.

[35] Nilsson PJ, van Etten B, Hospers GA, Påhlman L, van de Velde CJ, Beets-Tan RG, Blomqvist L, Beukema JC, Kapiteijn E, Marijnen CA, Nagtegaal ID, Wiggers T, Glimelius B (2013). Short-course radiotherapy followed by neo-adjuvant chemotherapy in locally advanced rectal cancer–the RAPIDO trial. BMC Cancer.

[36] Andre T, Boni C, Navarro M, Tabernero J, Hickish T, Topham C, Bonetti A, Clingan P, Bridgewater J, Rivera F, de Gramont A (2009). Improved overall survival with oxaliplatin, fluorouracil, and leucovorin as adjuvant treatment in stage II or III colon cancer in the MOSAIC trial. J Clin Oncol.

[37] André T, Boni C, Mounedji-Boudiaf L, Navarro M, Tabernero J, Hickish T, Topham C, Zaninelli M, Clingan P, Bridgewater J, Tabah-Fisch I, de Gramont A, Multicenter International Study of Oxaliplatin/5-Fluorouracil/Leucovorin in the Adjuvant Treatment of Colon Cancer (MOSAIC) Investigators (2004). Oxaliplatin, fluorouracil, and leucovorin as adjuvant treatment for colon cancer. N Engl J Med.

[38] Kuebler JP, Wieand HS, O'Connell MJ, Smith RE, Colangelo LH, Yothers G, Petrelli NJ, Findlay MP, Seay TE, Atkins JN, Zapas JL, Goodwin JW, Fehrenbacher L, Ramanathan RK, Conley BA, Flynn PJ, Soori G, Colman LK, Levine EA, Lanier KS, Wolmark N (2007). Oxaliplatin combined with weekly bolus fluorouracil and leucovorin as surgical adjuvant chemotherapy for stage II and III colon cancer: results from NSABP C-07. J Clin Oncol.

[39] Biagi JJ, Raphael MJ, Mackillop WJ, Kong W, King WD, Booth CM (2011). Association between time to initiation of adjuvant chemotherapy and survival in colorectal cancer: a systematic review and meta-analysis. JAMA.

[40] Lima IS, Yasui Y, Scarfe A, Winget M (2011). Association between receipt and timing of adjuvant chemotherapy and survival for patients with stage III colon cancer in Alberta, Canada. Cancer.

[41] Böckelman C, Engelmann B, Kaprio T, Hansen T, Glimelius B: **Risk of recurrence in patients with colon cancer stage II and III: A systemic review and meta-analysis of recent literature.** *Acta Oncol* In press 10.3109/0284186X.2014.975839 25430983

[42] Glimelius B (2014). Optimal time intervals between preoperative radiotherapy or chemoradiotherapy and surgery in rectal cancer?. Front Oncol.

[CR43] The pre-publication history for this paper can be accessed here:http://www.biomedcentral.com/1471-2407/14/948/prepub

