# Supplementary material for: A population-based cohort study on adherence to practice guidelines for adjuvant chemotherapy in colorectal cancer
Source: BMC Cancer. 2014 Dec 13;14:948. doi: 10.1186/1471-2407-14-948 (PMC4301907; doi:10.1186/1471-2407-14-948)
Supplement: Supplementary file 1 — Additional file 1: Table S1: Subgroup analyses in colon cancer stage II-III (n=12,726), 413 patients excluded due to missing data on planned adjuvant therapy. Patients remaining are 12,313. (DOC 92 KB) [file 12885_2014_5101_MOESM1_ESM.doc]

| **Additional file 1: Table S1.** Subgroup analyses in colon cancer stage II-III (n=12,726), 413 patients excluded due to missing data on planned adjuvant therapy. Patients remaining are 12,313. | | | | |
| --- | --- | --- | --- | --- |
|  | Proportion of patients planned for adjuvant chemotherapy | | | |
|  | Yes  (N) | Total*  (N) |  | (%) |
|  |  |  |  |  |
| **Stage II** | 875 | 6609 |  | 13.2 |
| Stage II, low risk | 135 | 3048 |  | 4.4 |
| Stage II, high risk^†^ | 738 | 3550 |  | 20.8 |
| Stage II, high risk, <50 years | 74 | 115 |  | 64.3 |
| Stage II, high risk, 50-59 years old | 142 | 272 |  | 52.2 |
| Stage II, high risk, 60-69 years old | 263 | 721 |  | 36.5 |
| Stage II, high risk, 70-75 years old | 184 | 714 |  | 25.8 |
| Stage II, high risk, 76-79 years old | 65 | 556 |  | 11.7 |
| Stage II, high risk, ≥80 years old | 10 | 1172 |  | 0.9 |
|  |  |  |  |  |
| Stage II, high risk, ≤ 75 years old, ASA 1 | 158 | 341 |  | 46.3 |
| Stage II, high risk, ≤75 years old, ASA 2 | 367 | 973 |  | 37.7 |
| Stage II, high risk, ≤ 75 years old, ASA 3 | 123 | 422 |  | 29.1 |
|  |  |  |  |  |
| Stage II, high risk, ≤ 75 years old, ASA 1, | 13 | 83 |  | 15.7 |
| no multidisciplinary conference |  |  |  |  |
| Stage II, high risk, ≤ 75 years old, ASA 2, | 36 | 226 |  | 15.9 |
| no multidisciplinary conference |  |  |  |  |
| Stage II, high risk, ≤ 75 years old, ASA 3, | 11 | 116 |  | 9.5 |
| no multidisciplinary conference |  |  |  |  |
|  |  |  |  |  |
| Stage II, high risk, ≤ 75 years old, ASA 1, | 145 | 257 |  | 56.4 |
| multidisciplinary conference |  |  |  |  |
| Stage II, high risk, ≤ 75 years old, ASA 2, | 331 | 746 |  | 44.4 |
| multidisciplinary conference |  |  |  |  |
| Stage II, high risk, ≤ 75 years old, ASA 3, | 112 | 306 |  | 36.6 |
| multidisciplinary conference |  |  |  |  |
|  |  |  |  |  |
| Stage II, high risk, ≤ 75 years old, female | 326 | 876 |  | 37.2 |
| Stage II, high risk, ≤ 75 years old, male | 337 | 946 |  | 35.6 |
|  |  |  |  |  |
| **Stage III** | 3455 | 5704 |  | 60.6 |
| Stage III, < 50 years | 271 | 282 |  | 96.1 |
| Stage III, 50-59 years old | 499 | 548 |  | 91.1 |
| Stage III, 60-69 years old | 1299 | 1470 |  | 88.4 |
| Stage III, 70-75 years old | 853 | 1127 |  | 75.7 |
| Stage III, 76-79 years old | 394 | 813 |  | 48.5 |
| Stage III, ≥ 80 years old | 138 | 1462 |  | 9.4 |
|  |  |  |  |  |
| Stage III, ≤ 75 years old, ASA1 | 740 | 786 |  | 94.1 |
| Stage III, ≤ 75 years old, ASA 2 | 1645 | 1863 |  | 88.3 |
| Stage III, ≤ 75 years old, ASA 3 | 452 | 646 |  | 70.0 |
|  |  |  |  |  |
| Stage III, ≤ 75 years old, ASA 1, | 136 | 149 |  | 91.3 |
| no multidisciplinary conference |  |  |  |  |
| Stage III, ≤ 75 years old, ASA 2, | 294 | 360 |  | 81.7 |
| no multidisciplinary conference |  |  |  |  |
| Stage III, ≤ 75 years old, ASA 3, | 77 | 125 |  | 61.6 |
| no multidisciplinary conference |  |  |  |  |
|  |  |  |  |  |
| Stage III, ≤ 75 years old, ASA 1, | 601 | 634 |  | 94.8 |
| multidisciplinary conference |  |  |  |  |
| Stage III, ≤ 75 years old, ASA 2, | 1347 | 1497 |  | 90.0 |
| multidisciplinary conference |  |  |  |  |
| Stage III, ≤ 75 years old, ASA 3, | 374 | 519 |  | 72.1 |
| multidisciplinary conference |  |  |  |  |
|  |  |  |  |  |
| Stage III, ≤ 75 years old, ASA 1-2, low grade | 1683 | 1858 |  | 90.6 |
| Stage III, ≤ 75 years old, ASA 1-2, high grade | 619 | 691 |  | 89.6 |
|  |  |  |  |  |
| Stage III, ≤ 75 years old, ASA 3, low grade | 315 | 453 |  | 69.5 |
| Stage III, ≤ 75 years old, ASA 3, high grade | 130 | 176 |  | 73.9 |
|  |  |  |  |  |
| Stage III, ≤ 75 years old, female | 1445 | 1656 |  | 87.3 |
| Stage III, ≤ 75 years old, male | 1477 | 1771 |  | 83.4 |
|  |  |  |  |  |
| *Includes patients planned and patients not planned for adjuvant chemotherapy. ^†^High-risk patients: clinical presentation with intestinal occlusion or perforation, lymph nodes sampling <12, pT4, poorly differentiated tumor (G3-G4), vascular, lymphatic or perineural invasion. | | | | |
